# Evaluation of a novel canine activity monitor for at-home physical activity analysis

**DOI:** 10.1186/s12917-015-0457-y

**Published:** 2015-07-04

**Authors:** Jonathan M. Yashari, Colleen G. Duncan, Felix M. Duerr

**Affiliations:** Department of Clinical Sciences, Veterinary Teaching Hospital, Colorado State University, Fort Collins, CO 80523 USA; Department of Microbiology, Immunology and Pathology, Colorado State University, Fort Collins, CO 80523 USA

**Keywords:** Physical activity monitoring, Accelerometer, Osteoarthritis, Gait analysis

## Abstract

**Background:**

Accelerometers are motion-sensing devices that have been used to assess physical activity in dogs. However, the lack of a user-friendly, inexpensive accelerometer has hindered the widespread use of this objective outcome measure in veterinary research. Recently, a smartphone-based, affordable activity monitor (Whistle) has become available for measurement of at-home physical activity in dogs. The aim of this research was to evaluate this novel accelerometer. Eleven large breed, privately owned dogs wore a collar fitted with both the Whistle device and a previously validated accelerometer-based activity monitor (Actical) for a 24-h time period. Owners were asked to have their dogs resume normal daily activities. Total activity time obtained from the Whistle device in minutes was compared to the total activity count from the Actical device. Activity intensity from the Whistle device was calculated manually from screenshots of the activity bars displayed in the smartphone-application and compared to the activity count recorded by the Actical in the same 3-min time period.

**Results:**

A total of 3740 time points were compared. There was a strong correlation between activity intensity of both devices for individual time points (Pearson’s correlation coefficient 0.81, p < 0.0001). An even stronger correlation was observed between the total activity data between the two devices (Pearson’s correlation coefficient 0.925, p < 0.0001).

**Conclusions:**

Activity data provided by the Whistle activity monitor may be used as an objective outcome measurement in dogs. The total activity time provided by the Whistle application offers an inexpensive method for obtaining at-home, canine, real-time physical activity data. Limitations of the Whistle device include the limited battery life, the need for manual derivation of activity intensity data and data transfer, and the requirement of Wi-Fi and Bluetooth availability for data transmission.

## Background

Accelerometers are small, light-weight, motion-sensing devices that record the intensity, frequency, and duration of movement for extended periods [1, 2]. Accelerometers have been used to quantify PA and energy expenditure in both humans and animals including dogs, cats and goats in the research and at-home environment [2–11]. A plethora of research evaluating the use of accelerometers in dogs is available including studies evaluating placement of the device [6, 11], ideal sampling period [12] and clinical validity [1, 13]. The Actical^1^ and other devices have been evaluated in several research studies, making it a well-validated device for PA monitoring in dogs [1, 2, 13, 14]. Despite this information, PA is infrequently used as an outcome measurement in canine clinical research [2]. This is likely related to the disadvantages of current accelerometers, mainly their cost and the time and inconvenience required for data evaluation. Furthermore, data recorded with current devices is limited to activity/energy expenditure data and does not allow for real-time monitoring.

A novel product by the name of Whistle^2^ has recently been introduced to the marketplace. This collar-bound product specifically designed for dogs contains a tri-axial accelerometer that measures PA. Tri-axial accelerometers measure motion in the vertical, mediolateral and craniocaudal planes [11]. The device is connected via Bluetooth to a tablet or smartphone and transmits data wirelessly over a Wi-Fi connection. Android and Apple applications are available for free download. The application allows dog owners to take notes, share photos and log the administration of medications or other events. This information would allow investigators to receive both subjective data from the owners while also acquiring objective, real-time information about the dogs’ at-home PA. These unique features, as well as the low cost of the device, make this device an attractive alternative to previously validated accelerometers.

The aim of this research was to compare the Whistle activity monitor for at-home PA monitoring in canines against a widely proven accelerometer (Actical). We hypothesized that the Whistle activity data would show strong correlation with the Actical data.

## Methods

Subjects - 11 dogs belonging to staff and students of the College of Veterinary Medicine and Biomedical Sciences at Colorado State University were recruited to participate in the study. Large-breed dogs greater than one year of age were included in the study. The study protocol was approved by the institutional animal care and use committee (Protocol ID: 15-5692A) and written owner consent was obtained.

**Fig. 1:**
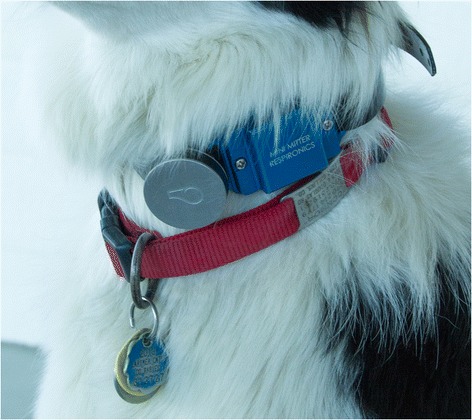
Whistle and Actical - Picture of a dog wearing the additional collar with the Whistle device (left) and Actical device (right) in its protective casing

Experimental Procedure – Activity in each dog was monitored by two accelerometers, the Actical^1^ and Whistle^2^ device, mounted side by side on a single nylon dog collar (Fig. 1). The same devices were used for each dog. The accelerometers were fastened on each dog so that both would be located ventrally on each dog’s neck as previously reported [6]. In order to protect the Actical device from both environmental and incidental damage while on the dog, the accelerometer was placed within a metal protective case provided by the manufacturer (Fig. 1). The accelerometer epoch (period of time where the device measure activity counts prior to saving it) was set at 1 min. The Whistle activity monitor was attached to the collar using the included rubber strap in addition to a zip-tie. The epoch length of the Whistle device is not adjustable but is preset by the manufacturer to 3 min.

Each dog had the collar adjusted to ensure a snug fit and owners were instructed to not place a leash on the collar used for attachment of the accelerometers. The owners were guided through the installation of the Whistle application on their respective smartphones and created a profile for their dog. The Whistle and owner’s smartphone were paired through both Wi-Fi and Bluetooth capabilities. The Bluetooth connectivity ensures communication between the Whistle device and smartphone even when the owner is not in range of a known Wi-Fi connection. Owners were asked to leave the collar on their dog continuously unless the dogs were planning on engaging in water activities to which they were asked to remove the collar. Data was collected while dogs were engaging in their typical activities.

**Fig. 2:**
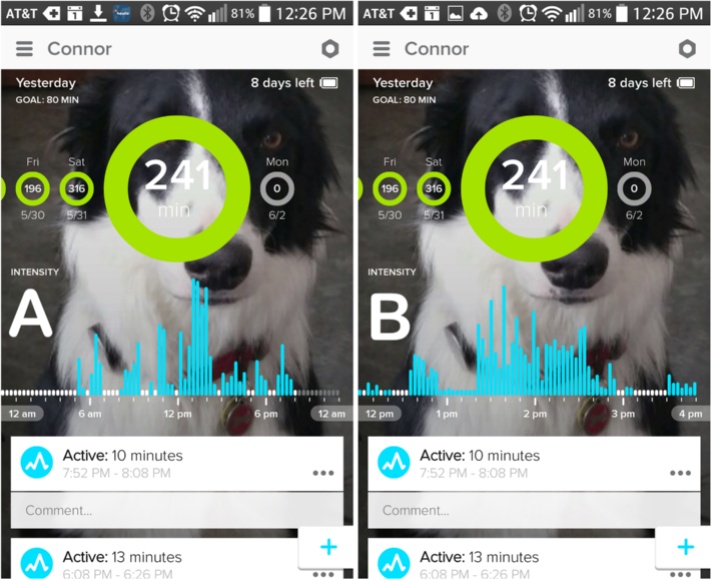
Enlarging the timeline on the Whistle application - Screenshots of the Whistle application display. **a** Activity bars displaying the entire day. **b** Activity bars displaying a 4 h time window after enlarging this time period

**Fig. 3:**
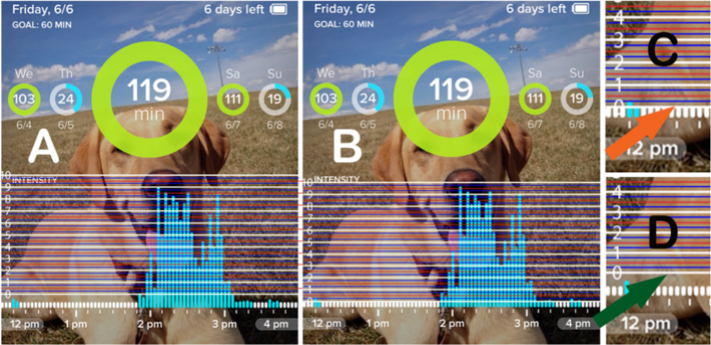
Measuring activity intensity using Whistle screenshots - Screenshots of the Whistle application display imported into Photoshop. **a** The grid for measurement of activity intensity is overlaying the activity bars in an incorrect position. **b** The grid is correctly aligned at the tip of the white ‘no activity’/white bars and at the bottom of the word ‘Intensity’. Enlargement of (**c**) correct and (**d**) incorrect alignment of the grid with the ‘no activity’ bars Activity bars

To access the Actical data, the device was removed from the collar and the device removed from the protective casing. The Actical was then connected to a PC using the provided Actical reader device. Data was downloaded using the proprietary, provided software. To access Whistle data, the authors created a secondary profile by adding an ‘owner’ to the profile of the dog currently wearing the collar. Activity intensity data was evaluated from 6:00 am to 11:00 pm while total activity data was evaluated over an entire 24 h period. Total activity data for the Actical (TAA) was defined as the total activity count during this time period. Total activity data for the Whistle (TAW) was defined as the total activity in minutes during the same day. Activity intensity for the Actical (AIA) was calculated by adding the activity counts for each three minute time period correlating to the Whistle timeline. In order to evaluate the activity intensity for the Whistle device (AIW), the smartphone application was used to display activity bars for each time period. Screenshots of the enlarged timeline (done by double-tapping on a time period, Fig. 2) were taken and then exported into a commercially available photo editing software (Adobe Photoshop, Adobe Systems, San Jose, CA). A total of five screenshots per dog were taken to include the time points of interest. A grid providing a scale of 0–10 with ¼ increments was created in Photoshop and then overlaid on each screen shot. The bottom of the grid was aligned with the top of the activity bars displaying ‘no activity’ (white bars); the top of the grid was aligned with the bottom of the word ‘Intensity’ (Fig. 3). Each activity bar (representing a 3 min time period) was then measured on the scale provided by the grid and assigned a value ranging from 0–10 (in ¼ increments) based on the tip of the blue bar for each respective time period.

Statistical analysis – Descriptive and comparative statistics were computed using SPSS 22^3^. Both Actical and Whistle outputs were explored visually as a group and by dog. Pearson’s correlation coefficients were calculated to assess for correlation between the intensity data (i.e. individual 3 min time intervals) as well as the total activity data.

## Results

Study participants included 7 castrated males and 4 spayed females (mean age: 4.13 ± 1.86 years, range: 1.5 – 7 years; mean weight: 30.2 ± 17.6 kg, range: 19–67 kg). Breeds included Alaskan Malamute (n = 2), Labrador Retriever Mix (n = 2), Border Collie Mix (n = 2), Border Collie (n = 2), Golden Retriever Mix (n = 1), Boxer Mix (n = 1), Border Collie Mix (n = 1). All dogs tolerated wearing the collar and for each of the 11 dogs in this study, data was collected simultaneously with both devices at 340 time points. This resulted in 3740 total time points for comparison. Output data is summarized in Table 1. For all dogs, there was a strong correlation between AIA and AIW (n = 3740, Pearson’s correlation coefficient 0.81, p < 0.0001) at all time points. The correlation for individual dogs ranged from 0.77-0.99 with only 2 dogs having a correlation coefficient less than 0.81. When time points in which no movement was detected in the Actical group were excluded the correlation was 0.78 (n = 2033, p < 0.0001). When evaluating the total activity data, there was a very strong correlation (Pearson’s correlation coefficient 0.921, p < 0.0001 between TAA and TAW).

**Table 1:** Summary of output data for total activity and activity intensity

	N	Minimum	Maximum	Mean	St. Dev.	Correlation
AIW	3740	0	9.75	0.70	1.52	0.81
AIA	3740	0	32044	987.40	2564.29	
TAW	11	79	317	127.82	67.67	0.93
TAA	11	102402	964737	335714.63	234370.06	

*TAW* total activity time Whistle, *TAA* total activity counts Actical, *AIW* activity intensity Whistle, *AIA* activity intensity Actical

## Discussion

Activity data provided by the Whistle device includes the total activity in minutes per day and a visual activity intensity display (Fig. 2). This study revealed a strong correlation for total activity obtained by the two accelerometers. TAW is a single number and can be recorded (but not exported) from the Whistle application. TAW is also emailed weekly to the ‘owners’ of a specific dog which may provide another means of obtaining this data in clinical research. We also found a strong correlation for activity intensity data between the two devices. However, the method used to derive the intensity data from the Whistle is time-consuming and cumbersome, limiting the use of this feature.

The Whistle device pairs with any smartphone/tablet via a free application allowing dog owners to create a profile for their dog, set daily PA goals, track their dogs PA, log food consumption, track medication administration (as well as receiving medication reminders), add photos, and enter notes throughout the day. The application also allows for multiple owners to log in, track, and manage activity through their mobile device. PA data collected from the Whistle is synced hourly with the owners’ smartphone or tablet which the Whistle is linked to. This eliminates the need for device-removal for data retrieval and allows real-time monitoring of PA. Real-time monitoring and the collection of objective and subjective data in one location offers a wide variety of opportunities for future research/clinical use. This may include tracking of post-operative clinical progression, response to specific treatments (since all data is ‘time-stamped’) and two-way communication with veterinarians including the sharing of pictures (such as for evaluation of wound healing etc.). Furthermore, measurement of joined activity between people and dogs is feasible using the Whistle device and Jawbone wristband. This may be of interest for evaluation of the impact of the human-animal bond and activity on both, human and dog health [15–17].

We chose the Actical activity monitor for comparison since it is a widely researched accelerometer that has been previously validated for the use in dogs [2–4, 6, 10, 11] and cats [5]. A recent study that evaluated the Actical in dogs found that there was 100 % specificity and 100 % sensitivity in distinguishing sedentary activity from walking activity and a 92 % specificity and 92 % sensitivity in distinguishing trotting activity from walking activity [4]. Another study found that the Actical is highly effective in differentiating sedentary activity from various degrees of activity in healthy dogs moving on a treadmill [11]. If the Whistle were to be used for activity intensity data collection, further investigations determining cut points between light, moderate, and vigorous activities may be indicated. Limitations of the Actical include its cost (at the time of writing priced at $450), the lack of real-time monitoring and the restriction to PA/energy expenditure measurement only. To access Actical data, the device must be physically removed from the protective housing/collar and placed onto the ActiReader, which must be connected to a PC. The data is then imported into the proprietary Actical Activity and Energy Expenditure analysis software and can be interpreted using this software or be exported into Microsoft Excel for further analysis. There are several disadvantages of the Whistle device including the lack of exportable data and battery life. As mentioned previously, to record TAW the researcher has to manually record this number either from the smartphone app or the weekly email sent to ‘owners’. The Whistle also uses proprietary software and the algorithms behind the data calculation are unknown. The battery life of the Whistle is approximately 7 days (compared to 240 days of the Actical), however, the device comes with a USB-charger for at-home use and charges within approximately 2 h. For long-term clinical studies the short battery life proposes a significant challenge for multiple reasons: Firstly, if owners forget charging their respective device, PA activity data recording is interrupted. Secondly, the dog’s activity during charging is not recorded. Lastly, while real-time data acquisition is a potentially useful feature, it eliminates the possibility of owner ‘blinding’ to their dog’s PA. Blinding would be feasible if owners were not allowed to pair their dog’s device, however, owner access to the smartphone app is necessary to check the device’s battery status. Since the battery lasts only a week, a study design where owners would be provided with a fully charged device would not allow for long-term outcome measurement. Furthermore, real-time monitoring is only feasible if the device is paired to the owner’s Wi-Fi network which requires some technical knowledge and a Bluetooth connection. This may limit participation of owners without Wi-Fi availability or the technical knowledge to perform the set-up.

The current study has several limitations. The Actical epoch length was set to 1 min whereas the Whistle is pre-set for an epoch length of 3 min. Since raw data was not available for the Whistle device, it is difficult to evaluate whether activities are recorded at the exact same moment. Data exploration suggested that less active dogs showed higher Whistle scores, however, this is difficult to confirm without availability of raw data. A second limitation is that all dogs participating in this study were large breed dogs. Therefore the results of this study may not apply to smaller dogs or cats. Only one device was used for this study, hence we can not comment on inter-device variability. Data evaluation for total activity data was performed over a 24-h time period. Previous studies have suggested a 7-day sampling interval for long-term clinical studies to account for differences in activity observed between week-days and weekend-days [12]. However, the purpose of this study was comparison of the two devices rather than evaluation of the study participants themselves. Lastly, data evaluation was only performed for a 17-h time period for the intensity data evaluation. However, this is consistent with previous canine [3] and human studies [18] and since each time point is evaluated individually, this should not affect our results.

## Conclusions

While it is not surprising that we showed strong correlation between the two accelerometers, such information is needed prior to using the Whistle device – or any other similar device - for clinical research. Total activity time provided by the Whistle application offers an inexpensive method to acquire real-time physical activity data in dogs. However, the limited battery life, need for manual data transfer, necessity of Wi-Fi and Bluetooth as well as the difficulty to obtain activity intensity data from the Whistle device may limit its use particularly for long-term studies.

## Abbreviations

TAW: Total activity time Whistle
TAA: Total activity counts Actical
AIW: Activity intensity Whistle
AIA: Activity intensity Actical
PA: Physical activity

## Footnotes

1 Animal Actical, Starr Life Sciences Corp., Oakmont, PA.

2 Whistle, Whistle Labs, San Francisco, CA (http://www.whistle.com).

3 (IBM Corp, Armonk, NY).
